# Effects of the Hormone Replacement Therapy and of Soy Isoflavones on Bone Resorption in Postmenopause

**DOI:** 10.3390/jcm7100297

**Published:** 2018-09-21

**Authors:** Delia Mirela Tit, Simona Bungau, Ciprian Iovan, Delia Carmen Nistor Cseppento, Laura Endres, Cristian Sava, Anca Maria Sabau, Gheorghe Furau, Cristian Furau

**Affiliations:** 1 Department of Pharmacy, Faculty of Medicine and Pharmacy, University of Oradea, 410028 Oradea, Romania; mirela_tit@yahoo.com (D.M.T.); simonabungau@gmail.com (S.B.); 2 Department of Preclinical Disciplines, Faculty of Medicine and Pharmacy, University of Oradea, 410073 Oradea, Romania; dr.iovan@biostandard.ro; 3 Department of Psycho Neuroscience and Recovery, Faculty of Medicine and Pharmacy, University of Oradea, 410073 Oradea, Romania; laura_endres@yahoo.com; 4 Department of Medical Disciplines, Faculty of Medicine and Pharmacy, University of Oradea, 410073 Oradea, Romania; cristian.sava2004@gmail.com; 5 Department of Physical Education, Sport and Physical Therapy, Faculty of Geography, Tourism and Sport, University of Oradea, 410087 Oradea, Romania; sabauancamaria@yahoo.com; 6 Department of Obstetrics and Gynecology, Arad County Clinical Hospital, 310048 Arad, Romania; gfurau@yahoo.com (G.F.); cristianfurau@gmail.com (C.F.); 7 “Vasile Goldis” Western University of Arad, 310045 Arad, Romania

**Keywords:** bone resorption, BMD, deoxypyridinoline, phytoestrogens, HRT

## Abstract

Postmenopausal osteoporosis is the most common form of osteoporosis and one of the major public health problems in developed countries. The prevalence of this condition, associated with the physiological stage of menopause, is continuously increasing. This study evaluated the effectiveness of soy isoflavones as compared to hormone replacement therapy (HRT) in low doses, on the prevention of postmenopausal osteoporosis, by determining bone mineral density (BMD) and urinary deoxypyridinoline (D-pyr) in physiological postmenopausal women. The study was conducted over a period of 12 months, on three parallel groups, which included a total of 325 postmenopausal women (HRT group: *n* = 95; phytoestrogens group: *n* = 124; control group: *n* = 106). At the one-year evaluation, we observed T-score normalization in a small number of cases (5.26%, 2.42% and 0.00%, respectively). The average values of D-Pyr decreased by 11.38% in the group treated with phytoestrogens (*p* < 0.05) and by 15.32% in the group that followed HRT (*p* < 0.05); it increased by 4.38% in the control group (*p* > 0.05). Both therapies have beneficial effects on bone metabolism, leading to a significant decrease in the evolution of bone resorption and there are no major differences between the efficacy of HRT and phytoestrogens in terms of the effects on BMD and bone resorption.

## 1. Introduction

Osteoporosis is a disease characterized by low bone mass and deteriorated micro-architecture of the bone tissue, leading to increased bone fragility and an increased risk of fracture [1]. This is a phenomenon especially connected with age and occurs frequently in postmenopausal women and elderly men [2].

According to the World Health Organization, osteoporosis is one of the major health problems in the developed world, occupying the second place as prevalence of the disease, due to female population aging [1,3,4]. The incidence of osteoporotic fractures is connected with the age of the population and the presence of skeletal and extra-skeletal risk factors [3,4,5]. The risk of occurrence of an osteoporotic fracture in women worldwide is 40% and, due to aging population, the global prevalence of osteoporotic fractures is expected to increase considerably [6,7]. Osteoporosis occurring after menopause interests particularly the trabecular bone and is maximal after 10 years of installation. During lifetime, women lose about 50% of the trabecular bone and about 30% of the cortical bone; about half is lost during the first 10 years after menopause [1].

The replacement of estrogen deficiency in postmenopausal with hormonal products slows the bone loss process by inhibiting the bone resorption, in the first years after the onset of the menopause, as well as in old age [8,9,10]. Due to the associated risks (venous thrombotic disease, breast cancer, stroke and coronary artery disease) [11,12], HRT is no longer the first choice in the prevention or treatment of osteoporosis; it is recommended only for women who have moderate to severe symptoms of menopause [10,13].

As a result, it has become imperative to seek alternative medicinal solutions that can be used to treat the same symptoms, but with fewer side effects. Research in recent years has brought attention to the role of phytoestrogens (selective estrogen receptor modulators) with beneficial effects in menopausal therapy and in maintaining health [14,15]. Various plant-derived estrogen-like composites have been categorized as selective estrogen receptor modulators by their nature; among them are included soy isoflavonoids, having raloxifene-like beneficial effects on the bones [14]. In epidemiological studies, it has been observed that women who frequently consume soy foods show lower risk of osteoporosis than women who have a classic Western diet [16,17,18]. Therefore, many menopausal women use phytoestrogens to maintain their bone mass (phytoestrogens are unlikely to cause those unwanted side effects associated with steroid hormones) [19].

The characteristic way in which isoflavonoids bind to estrogen receptors (RE), mainly beta-receptors (β-RE), makes them promising molecules for the replacement of hormones in therapeutic purposes [20]. The ability of isoflavones to mimic estrogen molecules in their ability to bind to RE is more pronounced in the case of genistein and daidzein and their derivative compounds [21]. These are the main isoflavones found in soybean, predominantly being daidzin, malonyldaidzin, genistin, and malonylgenistin (the glycosylated forms and, respectively, malonyl-derivatives of genistein and daidzein) [22]. Along with these substances, glycitein and glycitin (glycosylated form) are present in a smaller amount [22,23]. Genistein shows the greatest interest among isoflavones found in soy proteins [23].

Bone mass measurements are the most important predictor of fracture risk [24,25,26]. For this reason, World Health Organization guidelines (WHO) recommend that diagnosis of osteoporosis should be done using bone mineral density (BMD) measurements using X-ray bifunctional absorbance (DXA) [27]. For diagnosis, the measurement at the hip level is the gold standard regarding the spot as it has the highest predictive value for hip fracture, which is the most severe complication of osteoporosis and predicts the risk of all fractures as well as other techniques [28]. 

The change in bone mineral density is however reduced, postmenopausal bone loss after one year being approximately 2%. By DXA technique, lower rates of loss cannot be detected [29,30]. To evaluate the faster changes in the activity of the bones and the rate of bone loss during menopause, in recent years, the interest has focused on various biochemical markers of bone resorption [30,31]. A specific marker of the bone resorption is D-Pyr. It is released into the circulation during bone resorption process and excreted unchanged in the urine. Thus, the bone resorption induced by the menopause and the efficiency of antiresorptive therapy can be monitored by measuring the values of D-Pyr [30,31,32]. In research studies, D-Pyr has been used to reflect the increase of bone resorption induced in menopause as well as any reduction in bone resorption caused by HRT that results in slowing down or eliminating bone loss [33,34,35]. Research studies report correlations between high levels of D-Pyr and rapid bone loss as well as fracture risk. Measured in series, D-Pyr can help determine the alternative effect of bone resorption treatment, or any change caused by disease progression [28,35].

In this study, we compared the in vivo effectiveness of soy isoflavones (as alternative therapies to HRT, able to mimic the effects of the hormones that the body is no longer able to produce) with low-doses of HRT, in BMD evolution and in inhibition of the bone resorption in postmenopausal women, by monitoring the variations of BMD and D-Pyr values.

## 2. Experimental Section

### 2.1. Study Design

The study was conducted, during the period 2011–2014, in the County Clinical Emergency Hospital Oradea—Obstetric-Gynecological Ambulatory, and in private obstetrics–gynecology cabinets from Bihor County (NW Romania) on 325 postmenopausal women, divided into three groups. The first group was under HRT (1 mg estradiol and 0.5 mg NETA (norethisterone acetate) p.o. daily) and comprised 95 patients. The second group was treated with phytoestrogens (40% standardized extract with 20 mg soy isoflavones (genistein and daidzein), twp capsules, meaning 40 mg p.o. daily) and included 124 patients. The control group, without treatment, included 106 patients. 

The inclusion criteria in the study were physiological postmenopausal status (minimum one year and maximum five years) with vasomotor symptoms and no osteoporosis. The exclusion criteria were: women with induced menopause (surgical, chemotherapy or radiotherapy); menopause lasting over five years; hormone and phytoestrogen therapy; associated diseases that contraindicate HRT; chronic diseases that influence bone metabolism; and consuming supplements that could affect bone metabolism (calcium, vitamin D, etc.). The distribution of patients was performed using the selection method for each group, according to the patient’s willingness to take HRT or soy isoflavones, to the patient’s background, the assessment, the diagnosis, the risks, and the benefits of the indicated treatment [36]. The advantages of this study model imply the choice of therapy based on individual risks and patient choice. Clinical guidelines and therapeutic protocols provide that, before hormonal therapy is initiated, it is necessary for women to be informed by the physician that this therapy is associated with potential risks. It can be said that hormonal therapy is also a personal option; therefore, patients were not randomized to treatment.

The data were prospectively collected, stored and processed. We were interested in demographics data (age, origin), incidence of the risk factors of the osteoporosis, incidence of osteopenia, BMD correlated with the presence of risk factors, and the evolution of bone resorption by monitoring the variations of D-Pyr values.

After enrolling the patients, the study protocol consisted of baseline assessment, re-assessment at 6 months and 12 months after initiation of D-Pyr determination and at 12 months for BMD assessment by DXA technique. The study was conducted in accordance with the WMA Declaration of Ethical Helsinki—Medical Research Involving Human Principles for Subjects, approved by the Ethic Committee of the Faculty of Medicine and Pharmacy from the University of Oradea. Each patient included in this study signed an informed consent form before inclusion.

### 2.2. Clinical Investigations

BMD (grams per square centimeter) was measured with DXA and was expressed by T-score—the number of standard deviations with which bone density differs from the reference average for young adults. For women, four general diagnostic categories were proposed by the WHO and modified by the International Osteoporosis Foundation for DXA evaluations: normal *BMD*-T-score > −1; osteopenia (−1 < T-score < −2.5); osteoporosis-T-score ≤ −2.5; and severe osteoporosis-T-score ≤ −2.5 in the presence of one or more fragility fractures [24]. BMD was measured at the hip level. All measurements were performed using the same DXA machine—Prodigy Lunar Bone Densitometer (General Electric, Boston, MA, USA), at County Clinical Emergency Hospital Oradea.

Monitoring bone resorption was performed with IMMULITE 1000 System, using Pyrilinks-D Kit, for quantitative measurement of D-Pyr in urine by chemiluminescence immunoassay. D-Pyr determinations were made in a private laboratory in Oradea, at S.C. Biostandard. The reference range values for urinary D-Pyr, normalized to creatinine levels, was established by the laboratory at 3–6 nM D-pyr/mM creatinine. Urine samples were collected from the first urine, in sterile plastic containers, from all the women included in the study, and were stored at −20 °C till being processed.

### 2.3. Statistical Analysis

The statistical analysis was performed using EPIINFO, IBM SPSS Statistics 19, and MedCalc. All the average parameter values, standard deviations, frequency ranges, and statistical significance tests were calculated using the Student method (*t*-test) and χ^2^-test’s distribution was found to be similar to normal, being used by assumptions involving numerical data; paired *t*-test was the *t*-test used. Bravais–Pearson correlation coefficient was used to calculate an independent indicator of the units of measurement of the two variables. *p* < 0.05 value was attributed to statistical significance. ANOVA with a post-hoc analysis (Bonferroni) was used to analyze the differences between groups, as additional subgroup analysis.

## 3. Results

### 3.1. The Occurrence of Risk Factors for Osteoporosis

A statistical analysis of the data revealed that there was a uniform distribution of the patients, in the terms of both age and place of residence (urban or rural), without significant differences between all the three groups that were studied.

Risk factors for osteoporosis were identified in 49.49% of the patients comprised in the group treated by phytotherapy, 46.32% of those with HRT and 48.11% in the control group. The most common risk factor was the physical inactivity (44.35% for the group with phytotherapy, 42.11% for the group with HRT and 43.40% for the control group), followed by smoking (29.03% for the group with phytotherapy, 26.32% for the HRT group and to 29.25% for the control group) (Table 1).

### 3.2. Determination of T-Score (DXA) and Correlation with Risk Factors

At the initial assessment, depending on the diagnosis established at DEXA examination (BMD normal/osteopenia), the prevalence of osteopenia was 30.65% for the group treated with phytoestrogens, 31.58% for the HRT group and 33.02% for the control group (*p* = 0.612) (Table 2). 

The correlation of risk factors with the diagnosis established at the DEXA examination was performed for each study group (Table 3)

Regardless of the group, the prevalence of osteopenia is significantly higher in patients with risk factors for osteoporosis than in those without risk factors (44.26% vs. 17.46%, *p* < 0.001 in the phytoestrogens group, 45.45% vs. 19.61%, *p* < 0.001 in the THS group and 49.02% vs. 18.18%, *p* < 0.001 in the control group) 

### 3.3. Evolution of the Bone Mineral Density

At the 12-month evaluation, we recorded the following cases of normalization of the T-score: in the phytoestrogens group, three cases (2.42%); in the hormone therapy group, five cases (5.26%); and in the control group, no cases (0.00%). The incidence of osteopenia was 10.48% (13 cases) in the phytoestrogens group, insignificantly higher than in the hormone therapy group (8.42%, 8 cases) (*p* > 0.05) and insignificantly lower than in the control group 16.04%, 17 cases) (*p* > 0.05). Compared to the control group, the incidence of osteopenia is significantly lower in the hormone therapy group (*p* < 0.05). The incidence of osteoporosis was 3.23% in the phytoestrogens group, significantly higher than in the hormone therapy group (1.05%) (*p* < 0.05) and significantly lower than in the control group (13.21%) (*p* < 0.05). The incidence of osteoporosis was significantly lower in the hormone therapy group compared to the control group (1.05% vs. 13.21%) (*p* < 0.05) (Table 4). 

There were statistically significant differences in T-score evolution in all study groups, as determined by one-way ANOVA (F = 58.63876, *p* = 0.00001 for phytoestrogens group, F = 48.09626, *p* = 0.00001 for HRT group, and F = 35.68748, *p* = 0.00001 for control group). In the phytoestrogens group, the initial T-score had a mean of −0.343 and after 12 months the mean was −0.3118. Initially, in the same group, the T-score corresponding to osteopenia had a mean of −1.8105, reaching −1.7273 after 12 months of treatment. For HRT, the T-score of baseline corresponding to osteopenia had the baseline mean of −1.81 and reached −1.7781 after 12 months of treatment. For the control group (F = 35.68748, *p* = 0.001), T-score initially corresponding to osteopenia had the baseline mean of −0.3479 and reached −1.8105 after 12 months without treatment.

In patients with risk factors, there was no T-score normalization at 12 months in any of the groups. In contrast, in patients without risk factors, the T-score was normalized in three cases in the phytoestrogens group (4.76%), in five cases in the hormone therapy group (9.80%) (*p* > 0.05) and in no case in the control group (0.00%). The incidence of osteoporosis was 4.91% in the phytoestrogens group, insignificantly higher than in the HRT group (2.27%) (*p* > 0.05) and significantly lower than in the control group (15.69%, *p* < 0.05). In the groups with risk factors, the incidence of osteoporosis was significantly lower in the hormone therapy group than in the control group (2.27% vs. 15.69%) (*p* < 0.05).

In contrast, in patients without risk factors, the incidence of osteoporosis was 1.59% in the phytoestrogens group, significantly lower than in the control group (10.91%, *p* < 0.05). There was no case of osteoporosis in the group with hormone therapy of the group without risk factors (Table 5).

### 3.4. Bone Resorption Evaluation—DPD Determination in Urine

Values of D-Pyr over 6 nM/mM creatine were recorded at 56.45% for the group treated with phytoestrogens, 54.74% for the HRT group and 54.72% for the control group. The averages values of D-Pyr were between 6.16 recorded in the group treated with phytoestrogens and 6.19 in the control group, with no significant differences between the three groups (*p* > 0.05) (Table 6).

Assessment of D-Pyr values in patients with osteopenia, based on T-score, showed that values over 6 nM/ mM creatine were recorded at 81.58% for the group treated with phytoestrogens, 80.0% for HRT group and 82.86% for the control group. In patients without osteopenia, these rates were significantly lower: 45.35% of the group treated with phytoestrogens, 43.08% of the HRT group, and 40.85% of the control group. D-Pyr averages values in the cases with osteopenia were between 7.16 in the group treated with phytoestrogens and 7.23 in the control group, with no significant differences between the three groups (*p* > 0.05). In the cases without osteopenia, D-Pyr averages values were between 5.68 in the control group and 5.72 in the group treated with phytoestrogens (Table 7)

At six-month evaluation, the values of D-Pyr over 6 nM/mM creatine were recorded at 39.52% for the group treated with phytoestrogens, 35.79% for the HRT group, and 59.43% for the control group. The percentage of the patients with D-Pyr values > 6 nM/mM creatine decreased by 16.94% in the group treated with phytoestrogens and 18.95% in the HRT group, and increased by 4.72% in the control group. 

**Figure 1:**
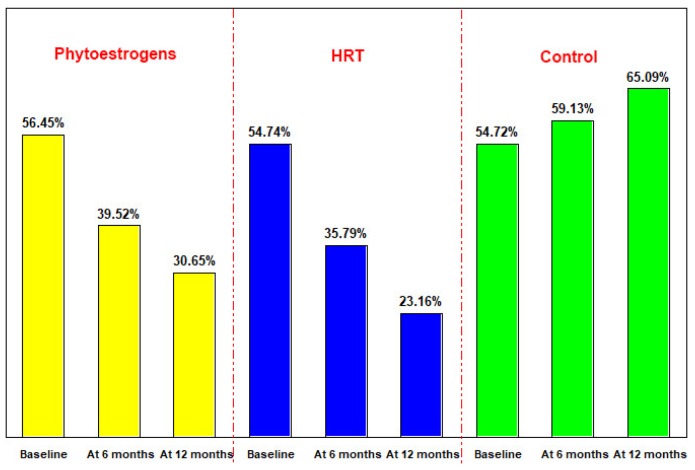
The distribution of cases according to D-Pyr values > 6 nM/mM creatine.

At 12 months, the values of D-Pyr over 6 nM/mM creatine were recorded at 30.65% for the group treated with phytoestrogens, 23.16% for the HRT group and 65.09% for the control group. The percentage of patients with D-Pyr values > 6 nM/mM creatine decreased in the groups treated with phytoestrogens or by HRT (respectively, 8.87% and 12.63%), and increased by 5.66% in the control group (Figure 1). Compared with the initial assessment, after 12 months, the percentage of patients with D-Pyr values > 6 nM/ mM creatine decreased by 25.81% in the group treated with phytoestrogens and 31.58% in the group with HRT, and increased by 10.38% in the control group. D-Pyr values were significantly different at all three measurements moments (baseline, 6 months and 12 months).

**Figure 2:**
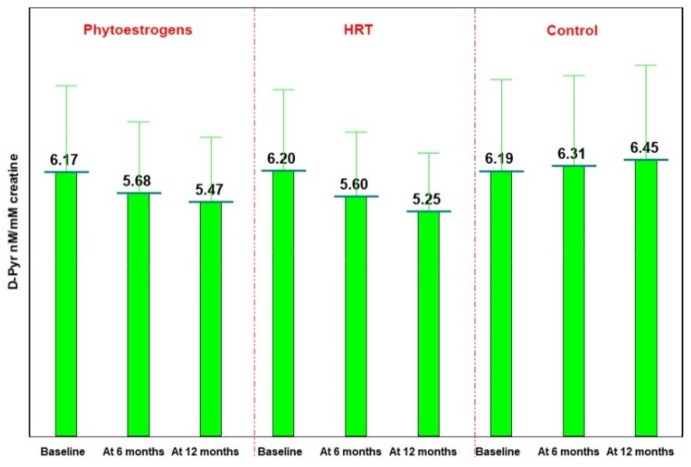
The evolution of averages values of D-Pyr.

There were found significant variations in D-Pyr in the groups treated with phytoestrogens and HRT, the average values decreasing by 11.38% in the phytoestrogens group (*p* < 0.05) and 15.32% in the HRT group (*p* = 0.035), and increasing by 4.38% in the control group (*p* > 0.05) (Figure 2).

ANOVA analysis revealed that at six months there were differences between the control group D-pyr levels compared to those treated with HRT, while at 12 months these differences became significant both between HRT and the control groups, as well as between phytoestrogen and control groups.

## 4. Discussion

Osteoporotic fractures are a significant cause of morbidity in postmenopausal women. For this reason, ways to prevent osteoporosis in this group of women is the focus of medical research. Effectiveness of estrogen therapy in preventing osteoporosis was demonstrated by numerous studies, but the risks of this therapy led to its retreating from the first-line therapies [37].

For this study, HRT was administered in small doses and phytoestrogens were administered in the form of standardized extract of soy isoflavones. To evaluate the effects of BMD therapy, the DXA technique was used and the evaluations were performed initially and at 12 months after the beginning of the treatment, the results being expressed by T-score (normal DMO/osteopenia/osteoporosis). According to literature data [28,37], the prevalence of osteopenia among young female population is 15% (T-score lower than −1) and it is expected that this percentage will increase at menopause. In our study, the prevalence of osteopenia was 30–33%, being much higher in women with risk factors (44–49%) than in those without (17–20%). These results are similar to the data from the literature [38,39]. Regarding the evolution of BMD, at the 12-month evaluation, we recorded the normalization of the T-score in a small number of cases (2.42%, 5.26% and 0.00%, respectively), all of these cases being without risk factors. The incidence of osteopenia and osteoporosis was minimal in the case of the hormone therapy group, insignificantly lower than in the phytoestrogens group but significantly lower than in the control group. Existence of risk factors led to a higher incidence of osteopenia and osteoporosis in all three groups.

To assess the bone changes during the treatment, we determined D-Pyr, an indicator of bone resorption induced by menopause and of the antiresorptive therapy efficiency [34,40], at the beginning of the study, after 6 and after 12 months. Comparing with the reference values, higher values of D-Pyr were recorded at approximately 56% of patients. This fact indicates an imbalance in bone remodeling process in more than half of menopausal women.

A longitudinal study [34] showed that D-Pyr values remain relatively constant in the years before menopause and begin to grow about six months after the last menstrual bleeding. The postmenopausal average values were 30–50% higher than the premenopausal average values for the same subjects. Other studies [41] on Caucasian women showed that menopause induced an 8.2% increase in the values of D-Pyr who returned to pre-menopausal levels within six months of HRT. Comparing the results of this study with the initial assessment, there a decrease in the percentage of patients with D-Pyr values > 6 nM/mM was observed, at the 6- and 12-month evaluations, for the HRT group and the group treated with phytoestrogens, which is consistent with other studies [42,43,44].

Supplementing estrogen deficiency after menopause by HRT slows the process of bone loss by inhibiting bone resorption, at both early post-menopausal and advanced age, as also demonstrated by this study. HRT is not the first choice in preventing or treatment of osteoporosis due to associated risks; it can only be considered in women with moderate to severe menopausal symptoms, osteopenia or early menopause [10]. THS contraindications are related to the fact that this therapy interferes with hepatic, cardiovascular pathology and increases the risk of estrogen-dependent cancers such as endometrial carcinoma or breast carcinoma [11,45]. In addition, therapy should be avoided in patients with undiagnosed genital hemorrhage, liver disease, or history of thromboembolic disorders [11], one of the major risks being thromboembolic events [11,46].

Selective estrogen receptor modulators (SERMs) are those compounds considered as postmenopausal alternative therapy; they improve bone function, having minimal risk in uterine and breast cancer cases [47]. Unfortunately, these substances are also associated with increased venous thromboembolic risk, as well as HRT, and have some unpleasant side effects, such as the intensification of hot flushes [48]. Isoflavones are substances that have been proposed as natural SERMs [48]. Their effects are mediated by estrogen receptor subtypes ERα and ERβ, and it has been demonstrated to be a dose-dependent and specific cell/tissue type [49].

In studies conducted on rodent or cell culture models, isoflavones are considered phytoestrogens that generally interact with ER and have poor estrogenic effect. These estrogen-like effects may raise soy or isoflavone consumption problems, especially in postmenopausal women at increased risk of breast cancer [19]. These concerns are not supported by high intakes of soya and soy products (the main source of dietary phytoestrogens); these levels have been observed in epidemiological studies according to which they are associated (in both sexes) with low rates of cancer, cardiovascular disease and osteoporosis, and in postmenopausal women are associated with a low number of hot flushes [50,51]. There is relatively little current evidence to confirm that this potential weak estrogenic effect of dietary isoflavones has a significant clinical impact on breast tissue in healthy women. Few scientific data indicate that this is true also for survivors of breast cancer [50,51,52,53,54]. There are studies that highlight the fact that isoflavones have antioxidant activity in vitro and exert antiproliferative activities. The molecular endocrinology of phytoestrogens and SERMs is very different, so there is only a weak association between SERM action and phytoestrogens [48]. 

In different cases, women’s health needs should be evaluated on the basis of a multidimensional assessment of their physical, mental, and social well-being. Phytoestrogens are therefore thought to be potentially a significant contributor to non-steroidal (dietary origin) estrogens with health effects, particularly relevant for patients (women) at risk for hormone-associated diseases.

## 5. Conclusions

BMD expressed by T-score showed favorable treatment progression in patients treated with HRT and phytoestrogens, but without significant T-score variations. The D-Pyr values for the women included in the control group were significantly different from those included in the therapy groups. In addition, the D-Pyr values for the women treated with HRT were not significantly different from those obtained for the women treated with phytoestrogens.

Both therapies, the hormone and phytoestrogens, have beneficial effects on the bone metabolism, causing a significant decrease in bone resorption process. Comparative assessment showed no significant differences between the effectiveness of the hormone therapy and the phytoestrogens used in the study, in terms of effects on BMD and bone resorption, when administered to groups of women with the same sociodemographic and clinical characteristics.

## Figures and Tables

**Table 1:** The distribution of cases depending on the osteoporosis risk factors.

Risk Factors	Groups					
	Phytoestrogens		HRT		Control	
	No.	%	No.	%	No.	%
**With risk factors**	61	49.19	44	46.32	51	48.11
Smoking	36	29.03	25	26.32	31	29.25
Alcohol	13	10.48	7	7.37	9	8.49
Sedentariness	55	44.35	40	42.11	46	43.40
Corticosteroids	5	4.03	3	3.16	3	2.83
Fracture in history	8	6.45	7	7.37	7	6.60
Rheumatoid arthritis	3	2.42	1	1.05	3	2.83
FHH * osteoporosis/fractures	10	8.06	11	11.58	12	11.32
**Without risk factors**	63	50.81	51	53.68	55	51.89

* Family Health History.

**Table 2:** The distribution of cases depending on T-score.

BMD	Groups					
	Phytoestrogens		HRT		Control	
	No.	%	No.	%	No.	%
Normal BMD (T-score > −1)	86	69.35	65	68.42	71	66.98
Osteopenia (−1 < T-score < −2.5)	38	30.65	30	31.58	35	33.02

**Table 3:** Distribution of cases by T-score depending on the presence of risk factors.

BMD	Phytoestrogens		HRT		Control	
	No.	%	No.	%	No.	%
**With risk factors**						
Normal BMD (T-score > −1)	34	55.74	24	54.55	26	50.98
Osteopenia (−1 < T-score < −2.5)	27	44.26	20	45.45	25	49.02
**Without risk factors**						
Normal BMD (T-score > −1)	52	82.54	41	80.39	45	81.82
Osteopenia (−1 < T-score < −2.5)	11	17.46	10	19.61	10	18.18

**Table 4:** Evolution of cases according to T-score.

BMD	Phytoestrogens		HRT		Control	
	No.	%	No.	%	No.	%
**Baseline evaluation**						
Normal	86	69.35	65	68.42	71	66.98
Osteopenia	38	30.65	30	31.58	35	33.02
Osteoporosis	0	0.00	0	0.00	0	0.00
**Evaluation at 12 months**						
Normal	76	61.29	62	73.68	54	50.94
Osteopenia	44	35.48	32	33.68	38	35.85
Osteoporosis	4	3.23	1	1.05	14	13.21

**Table 5:** Evolution of cases according to T-score and risk factors.

BMD	With Risk Factors				Without Risk Factors			
	Baseline		At 12 Months		Baseline		At 12 Months	
	No.	%	No.	%	No.	%	No.	%
**Phytoestrogens group**								
Normal	34	55.74	25	40.98	52	82.54	51	80.95
Osteopenia	27	44.26	33	54.10	11	17.46	11	17.46
Osteoporosis	0	0.00	3	4.92	0	0.00	1	1.59
**HRT group**								
Normal	24	54.55	18	40.91	41	80.39	44	86.27
Osteopenia	20	45.45	25	56.82	10	19.61	7	13.73
Osteoporosis	0	0.00	1	2.27	0	0.00	0	0.00
**Control group**								
Normal	26	50.98	15	29.41	45	81.82	39	70.91
Osteopenia	25	49.02	28	54.90	10	18.18	10	18.18
Osteoporosis	0	0.00	8	15.69	0	0.00	6	10.91

**Table 6:** The distribution of cases according to D-Pyr values (nM/mM creatine) at the base line.

D-Pyr Values (nM/mM Creatine)	Groups					
	Phytoestrogens		HRT		Control	
	No.	%	No.	%	No.	%
3–3.9	8	6.45	7	7.37	9	8.49
4–4.9	18	14.52	16	16.84	13	12.26
5–5.9	28	22.58	20	21.05	26	24.53
6–6.9	38	30.65	23	24.21	27	25.47
7–7.9	23	18.55	20	21.05	21	19.81
≥8	9	7.26	9	9.47	10	9.43
M ± SD	6.16 ± 2.01		6.20 ± 1.89		6.19 ± 2.13	

**Table 7:** The distribution of cases according to D-Pyr values (nM/mM creatine) and depending on T-score.

D-Pyr (nM/mM Creatine)	Groups					
	Phytoestrogens		HRT		Control	
	No.	%	No.	%	No.	%
**With osteopenia**						
3–3.9	0	0.00	0	0.00	0	0.00
4–4.9	2	5.26	2	6.67	3	8.57
5–5.9	5	13.16	4	13.33	3	8.57
6–6.9	9	23.68	5	16.67	8	22.86
7–7.9	14	36.84	12	40.00	12	34.29
≥8	8	21.05	7	23.33	9	25.71
M ± SD	7.16 ± 2.32		7.22 ± 2.41		7.23 ± 2.33	
**Without osteopenia**						
3–3.9	8	9.30	7	10.77	9	12.68
4–4.9	16	18.60	14	21.54	10	14.08
5–5.9	23	26.74	16	24.62	23	32.39
6–6.9	29	33.72	18	27.69	19	26.76
7–7.9	9	10.47	8	12.31	9	12.68
≥8	1	1.16	2	3.08	1	1.41
M ± SD	5.72 ± 1.64		5.70 ± 1.52		5.68 ± 1.81	
